# Development and validation of students’ digital competence scale (SDiCoS)

**DOI:** 10.1186/s41239-022-00330-0

**Published:** 2022-05-16

**Authors:** Katerina Tzafilkou, Maria Perifanou, A. A. Economides

**Affiliations:** 1 SMILE Lab, University of Macedonia, Egnatia 156, 54636 Thessaloníki, Greece; 2 SMILE Lab & Information Systems IPPS, University of Macedonia, Thessaloníki, Greece

**Keywords:** Adult learning, Digital competence, Digital skills, Scale development, Twenty-first century skills

## Abstract

Towards the transition to blended and remote education, evaluating the levels of students’ digital competence and designing educational programs to advance them is of paramount importance. Existing validated digital competence scales usually ignore either important digital skills needed or new socio-technological innovations. This study proposes and validates a comprehensive digital competence scale for students in higher education. The suggested instrument includes skills of online learning and collaboration, social media, smart and mobile devices, safety, and data protection. The scale was evaluated on a sample of 156 undergraduate and postgraduate students just before and at the beginning of the COVID-19 crisis. The final scale is composed of 28 items and six digital competence components. The evaluation study revealed valid results in terms of model fit criteria, factor loadings, internal validity, and reliability. Individual factors like the students’ field of study, computer experience and age revealed significant associations to the scale components, while gender revealed no significant differences. The suggested scale can be useful to the design of new actions and policies towards remote education and the digital skills’ development of adult learners.

## Introduction

New digital trends and technologies are reshaping the way people work, communicate, and learn. According to the OECD report (OECD Skills Outlook, 2019; p. 11), “Countries’ preparedness to seize the benefits of digital transformation is largely dependent on the skills of their populations …” Today such skills are even more critical for teachers and students due to the COVID-19 crisis and the context of Emergency Remote Education (ERE). During the COVID-19 ERE transition, teachers and students shifted to fully online teaching and learning (OECD, 2019). The shift to ERE is heavily dependent on the individuals’ digital skills; hence evaluating their digital competence might be practically useful for educational institutions, pedagogy designers, and educational policy makers towards the design of efficient ERE strategies. Although recent studies have evaluated the usefulness of the educational technologies used in the context of ERE (Bond et al., 2021), the research on students’ digital skills or online readiness is still limited.

Digital competence (DC) traditionally reflects a person’s ability to use digital technologies in a critical, collaborative, and creative way; also, the person should have the knowledge, skills, and attitude to be perceived as having the competence on a domain (European Commission, 2019a; Marusic & Viskovic, 2018; Suwanroj et al., 2017, 2018). A student’s perceived digital competence reflects his/her Information and Communication Technologies (ICT)-based knowledge and skills that can be used to perform ICT-related tasks (Meng et al., 2019). Recent works confirm that students’ perceived ICT competence significantly affect their academic achievement (Park & Weng, 2020) and highlight the importance of understanding the global ICT trends on mobile, Internet and social media use (We Are Social & Hootsuite, 2020). The European Commission (2020) also reports that such skills of social media and mobile use should be included in the Digital Competence and New Skills Agenda.

Research shows that there are several ‘barriers’ in supporting young adults’ digital skills development; such barriers include the poor access to technology and limited support networks (Eynon & Geniets, 2016). The authors also explain that lack of experience and of digital skills decreases the levels of perceived usefulness of Internet in young people’s lives. Also, according to Cullinan et al. (2021), one-in-six higher education students are at risk of poor access to Internet, posing a significant barrier to attend their courses during the pandemic. The European Commission (EC, 2018a) admits that there is an urgent need to speed up the exchange of good practices in the field of adult digital education.

Attempting to measure and quantify the students’, teachers’ or citizens’ digital skills, several studies have developed methodologies to identify the key components of digital competence (e.g., All Aboard!, 2015; European Commission, 2019a). The newest version of the European Digital Competence Framework (DigComp 2.0) describes which skills are required to use digital technologies “in a confident, critical, collaborative and creative way to achieve goals related to work, learning, leisure, inclusion and participation in our digital society” (European Commission, 2019a). Several other frameworks suggest different versions (e.g., ESCO, 2019; Fraillon et al., 2019; UNESCO, 2018) of a digital competence framework, while recent studies attempt to extend the previous DC scales by including contemporary skills of critical thinking, communication, etc. (Peart et al., 2020).

However, these studies mainly concern the generic population and are not student oriented. Most important, the recently emerged digital skills regarding mobile/e-learning, mobile/e-commerce and social media activities are considered in a limited number of studies (e.g., Perifanou, & Economides, 2019b; Lee et al., 2015). Last, several research studies are focused on measuring the students’ digital skills across different contexts and regions using existing students’ DC frameworks, but only a few (e.g., Alarcón et al., 2020; Kuzminska et al., 2018) attempted to quantitatively evaluate or adjust the applied scales.

Motivated by the afore described research gap, this study seeks to quantitatively adjust and evaluate a recent instrument on the students’ DC, forming a validated students’ digital competence scale (SDiCoS) that can be applied in the context of remote education and university students. The suggested validated scale is based on a recently proposed framework and instrument (Perifanou, & Economides, 2019a, 2019b) which aims at measuring individuals’ digital skills and knowledge on today’s computer and Internet use, as well as social media and mobile activities. Also, since previous studies reported the effects of personal factors on students’ digital skill components (He & Zhu, 2017; Tømte & Hatlevik, 2011) and online learning (Yu, 2021), this study also seeks to explore the potential differences across the DC components based between different groups of students. Towards this goal, the main research objectives (ROs) are formed as follows:

- RQ1: To develop and quantitatively validate a scale to measure the students’ digital competencies considering the context of remote education.
- RQ2: To explore the significant differences in the students’ digital skills, between different groups of students including their gender, age, field of study and experience in computer use.

Overall, the findings can contribute towards the design of a comprehensive DC scale that considers recent technological trends, and concerns both undergraduate and postgraduate students’ competence items. Also, it might be practically useful towards the design and implementation of actions or policies to detect DC gaps and reinforce the adult learners’ digital competence in remote and blended learning.

## Related works

Several previous studies examined the structure of digital competence models and instruments by applying statistical methods. Many of those studies (e.g., Oberländer et al., 2020; Tondeur et al., 2017; Touron et al., 2018) performed first and/or second order confirmatory factor analyses (CFA). Other studies performed exploratory factor analyses (EFA) to identify the main components that form digital competence scale (e.g., Internet skills scale, Technology/ICT Literacy, etc.) either for students’ (e.g., Lau & Yuen, 2014; van Deursen et al., 2016) or teachers’ digital skills (e.g., Siddiq et al., 2016; Touron et al., 2018).

Furthermore, much of the research in students’ DC regards the examination of structural relationships between the components (e.g., Aesaert et al., 2015; Hatlevik et al., 2015; Schmid & Petko, 2019) or it has been implemented out of the educational context, mainly focusing on the employment sector (e.g., Oberländer et al., 2020).

Table 1 selectively presents the scale size, components, and validation methods of previous quantitative studies that designed DC scales (either for students, teachers, or other individuals), in the context of higher, secondary, or primary education across different regions.

**Table 1:** Quantitative studies on digital skills scale development/validation across different regions

Authors	Method(s)	Software	Scale/components	Target subject/Country
Alarcón et al. (2020)	CB-CFA and correlation analysis	N/A	8 components-29 items (1) Professional engagement, (2) Digital resources, (3) Teaching and learning, (4) Assessment, (5) Empowering, (6) Facilitating learners’ digital, (7) Digital environment, and (8) Extrinsic digital engagement	Educators/Spain and Latin America
Peart et al. (2020)	EFA and CB-CFA	N/A	11 components-59 items (1) Management and use of information and data, (2) Communication skills, (3) Digital content creation, (4) Management and security of information and digital content, (5) Ethics and digital responsibility, (6) Social and political behaviours and attitudes, (7) Digital empathy, (8) Social and digital engagement, (9) Critical thinking, (10) Democratic attitudes, (11) Prosocial behaviour	Young people/Spain, United Kingdom
Suwanroj et al. (2019)	2nd order CFA	LISREL 8.72	7 components-24 items (1) Fundamental of digital, (2) Accessing digital information, (3) Using digital information, (4) Creating digital information and media, (5) Communicating digital information, (6) Managing digital information, and (7) Evaluating digital information	Undergraduate students/Thailand
Kong et al. (2019)	EFA and CB-CFA	SPSS 21, AMOS 24	4 components-16 items (1) Meaningfulness, (2) impact, (3) Creativity belief, and (4) Competence belief	Primary school pupils/Hong Kong
Blayone et al. (2018)	Correlation analysis and Cronbach alpha	N/A	4 components-26 items (1) Technical, (2) communicational, (3) Informational, Computational	Students (higher education)/Georgia and Ukraine
Kim and Choi (2018)	EFA and CB-CFA	AMOS 23.0	5 components-17 items (1) Self-identity in digital environment, (2) Reasonable activity online, (3) Social/cultural engagement, (4) Fluency for the Digital tools, and (5) Ethics for digital environment	Teachers/Korea
Touron et al. (2018)	1st and 2nd order CB-CFA	AMOS 23	5 components-54 items (1) IT information and literacy information, (2) Communicating and collaborating, (3) Creating digital content, (4) Security, and (5) Troubleshooting	Teachers/Spain
Kuzminska et al. (2018)	EFA	SPSS	2 components-18 items (1) Digital competencies as mean of communication, and (2) competencies of professional usage digital resources	Students and teachers (higher education)/Ukraine
Tondeur et al. (2017)	EFA and CB-CFA	SPSS 21, AMOS 21	2 components-19 items (1) Competencies to support pupils for ICT use in class, and (2) Competencies to use ICT for instructional design	Pre-service teachers/Belgium
Elstad and Christophersen (2017)	CB-SEM CFA	SPSS AMOS 22	2 components-16 items (1) Self-efficacy for influencing students’ use of ICT in the service of learning, and (2) Self-efficacy for maintaining discipline	Students and teachers (higher education)/Norway
Choi et al. (2017)	EFA and CFA	AMOS	5 components-26 items (1) Internet political activism, (2) Technical skills, (3) Local/global awareness, (4) Critical perspective, and (5) Networking Agency	Students (under and postgraduate)/USA
Al Khateeb (2017)	EFA and correlation analysis	N/A	4 components-19 items (1) Procedural competence, (2) Social-digital competence, (3) Digital discourse and (4) Strategic competence	Preservice teachers/Kingdom of Saudi Arabia
Mengual-Andrés et al. (2016)	Correlation analysis and Cronbach alpha	N/A	5 components-52 items (1) Technological literacy, (2) Information access and use, (3) Communication and collaboration, (4) Digital citizenship, (5) Creativity and innovation	Students (higher education)/Spain
Siddiq et al. (2016)	ESEM	MPLUS 7.2	3 components-12 items (1) Accessing digital information, (2) Evaluating digital information, (3) sharing and communicating digital information	Teachers (secondary education) / Norway
Koc and Barut (2016)	EFA and CB-CFA	SPSS	4 components-35 items (1) Functional consumption, (2) Critical consumption, (3) Functional prosumption, and (4) Critical prosumption	Students (higher education)/Turkey
van Deursen et al. (2016)	EFA and CB-CFA	AMOS	5 components-35 items (1) Operational skills, (2) Navigation information skills, (3) Social skills, (4) Creative skills, (5) Mobile skills	General public (students: 16.3%)/UK and Netherlands
Lee et al. (2015)	EFA and CB-CFA	N/A	2 components-8 items (1) Participation and distribution, and (2) Creation and production	Students (primary, secondary, and junior college)/Singapore
Hatlevik et al. (2015)	Correlation analysis and multilevel analysis	SPSS 21	5 components-25 items (1) Cultural capital, (2) Language integration, (3) Previous academic achievements, (4) Self-efficacy, and (5) Strategic information use	Secondary students/Norway
Aesaert et al. (2015)	EFA and SEM-CFA	AMOS 21	7 components-94 items (1) Learning motivation, (2) Learning style, (3) Parental ICT attitude, (4) Parental ICT support, (5) Teacher’s ICT attitude, (6) Pupil’s ICT attitude, and (7) ICT self-efficacy	Primary school pupils/Belgium
Lau and Yuen (2014)	EFA and CB-CFA	AMOS 20.0.0	3 components-17 items (1) Information literacy, (2) Internet literacy, and (3) Computer literacy	Secondary school students/Hong Kong

*CB* Covariance-based, *CFA* Confirmatory Factor Analysis, *EFA* Exploratory Factor Analysis, *ESEM* Exploratory Structural Equation Modelling, *SEM* Structural Equation Modelling

As depicted in Table 1, only a few studies have been validated in the population of undergraduate students and/or in European countries. Second, none of the cited studies has employed a partial least squares structural equation modeling (PLS-SEM) approach to identify or confirm a digital competence measurement scale, although PLS-SEM has been proved more reliable for applying confirmatory factor analyses, compared to Covariance-based (CB-SEM) approaches (Asyraf & Afthanorhan, 2013).

In the meanwhile, there have been several studies (Marusic & Viskovic, 2018; Suwanroj et al., 2017, 2018) that examined the structure of digital competence instruments by applying qualitative approaches (e.g., expert views and/or combined/review-based approaches). Recently, Perifanou and Economides (2019a, 2019b) proposed a comprehensive framework and an instrument consisting of 56-items to measure the students’ DC. The suggested instrument is informed by and extends previous popular DC frameworks (All aboard!, 2015; European Commission, 2019a; Fraillon et al., 2019; UK, 2019; UNESCO, 2018). Comparing to previous models, this instrument meets the today’s DC requirements by including skills related to the social media and mobile use.

## Materials and methods

### Instrument

The initial instrument of this study (Perifanou, & Economides, 2019a, 2019b) was composed of 56 items and four dimensions namely (i) Access, Search and Find, (ii) Use, Store, Manage, Evaluate and Delete, (iii) Communicate, Collaborate, and Share, and (iv) Create, Apply, Modify, Combine, Solve and Protect. The items in the four dimensions considered new digital innovations (e.g., social media and smart devices), as well as ethical and responsible behavior).

For the needs of this study, some items were adjusted through rephrasing or adding explanatory comments and examples. Five experts in the field of Technology Enhanced Learning (TEL) reviewed the instrument’s items regarding the wording, and the quality of the items, to minimize misperceptions. Then, after an initial PLS-SEM evaluation of the responded adjusted questionnaire, several items were removed due to low internal consistency scores, forming at last a six-component (by adding two components) instrument and 28 items. So, the initial 4 dimensions were adjusted to six components and the initial 56 items were reduced to 28 items. The proposed components are (1) Search, Find, Access (SFA); (2) Develop, Apply, Modify (DAM); (3) Communicate, Collaborate, Share (CCS); (4) Store, Manage, Delete (SMD); (5) Evaluate (EV); and (6) Protect (PR). The final instrument is presented in Appendix. All the items are measured on a 5-point Likert scale (1: strongly disagree to 5: strongly agree). The questionnaire’s used terms were explained to the participants as follows: “Smart device = smartphone, tablet, laptop, pc, camera, navigator, game console, smart TV, etc.; Object = document, picture, movie, software, app, etc.”.

### Sample characteristics and data collection

During the period between January and February 2020, the DC questionnaire was distributed in a written form to students in two different undergraduate university courses (e-Commerce and e-Business, Information Systems in Management), and in April 2020 it was sent out online in three postgraduate programmes (Information Systems, e-Business & Digital Marketing, Law & Economics) in Greece. The second part of the survey (April, 2020) was conducting within the COVID-19 crisis and the school closure in Greece, hence all participants were already attending emergency remote courses. The remote courses were conducted through synchronous video lectures via the Zoom platform, and the courses’ materials were uploaded to the Open eClass online platform for asynchronous education. Open eClass is an open-source integrated e-course management system compatible with the international standard Sharable Content Object Reference Model (SCORM).

The questionnaire items were measured on a five-point Likert scale from “Strongly disagree” to “Strongly agree”. The questionnaire also asked for some social and academic information (gender, age, experience in mobile and computer use, average grade in last semester, etc.). The total population that was invited to participate in the survey voluntarily and anonymously was 300 students.

All participants were asked to consent for their volunteer and anonymous participation in the study. It was not possible to identify the identity of any respondent and all ethics standards were met according to the university internal committee. Several students did not complete the questionnaire and after eliminating the invalid answers the final working sample was 156 students, 80 undergraduates and 76 postgraduates. The respondents’ socio demographic characteristics are presented in Table 2.

**Table 2:** Respondents socio-demographic characteristics (N = 156)

Gender	n%	Age	n%	Study programme	n%	Computers use experience (in years)	n%
Female	56.4	18–24	59	e-Commerce and e-business (undergraduate)	14.7	1–5	3.8
Male	42.9	25–35	29.5	Information systems in management (undergraduate)	36.5	6–10	45.5
N/A	0.6	36–45	7.1	e-Business and digital marketing (postgraduate)	14.1	11–20	47.4
		46–55	2.6	Law and economics (postgraduate)	8.9	> 20	3.2
		55+	1.9	Information systems (postgraduate)	19.2		
				Undefined	6.4		

### Data analysis

Structural Equation Modelling (SEM) is considered as one of the most important statistical developments in social sciences (Hair et al., 2011). SEM elaborates in a comprehensive and efficient manner the relationships among multiple independent and dependent constructs (the structural model) simultaneously (Gefen et al., 2000; Hair et al., 2010). Moreover, SEM not only assesses the structural model but also evaluates the measurement model (Gefen et al., 2000, 2011). Researchers applying SEM can choose between a covariance base analysis (CB-SEM) or partial least squares (PLS-SEM) (Gefen et al., 2000; Hair et al., 2011). Recently researchers introduced methods that provide consistent PLS-SEM estimations that can be used complementary or alternatively to CB-SEM (Bentler & Huang, 2014; Dijkstra, 2014; Dijkstra & Henseler, 2015).

Contrary to previous studies in the literature that used mainly CB-SEM approaches, this study applied a hybrid PLS-SEM and CB-SEM approach to evaluate the suggested scale, in terms of internal consistency, composite reliability, convergence validity and discriminant validity. PLS-SEM was applied for the following reasons:

- According to the suggestions of Bentler and Huang (2014), Dijkstra (2014), and Dijkstra and Henseler (2015) who proved that PLS-SEM can consistently mimic common CB-SEM approaches, PLS-SEM is an appropriate approach to study and validate the structure of a model. In this study, the primary scale validation is based on PLS-SEM CFA mainly because of the non-normality observed in the data (Shapiro & Wilk, 1965), the small sample size (Hair et al., 2014), and the adequateness of the method compared to CB-based approaches, as suggested in Asyraf and Afthanorhan (2013) and Rigdon (2012).
- Furthermore, as recommended by Hair et al. (2011; p.144) a PLS-SEM approach should be implemented if “the goal is predicting key target constructs or identifying key ‘driver’ constructs” or if research is exploratory or an extension of an existing structural theory”. Contrary, a CB-SEM approach should be chosen if “the goal is theory testing, theory confirmation or comparison of alternative theories”. Although many researchers focus on comparing the differences of model estimations when using CB-SEM and PLS-SEM, both methods are complementary rather than competitive.

Based on the above, our methodological approach was based on the following steps:

i. A PLS-SEM CFA was applied to primarily test for the model structure validation, using the software SmartPLS;
ii. A CB-based CFA replication was applied, using Amos software, to further examine the results of factor loadings and model fit values;
iii. A second-order CFA was conducted, using Amos software, to further validate the results and examine whether a broad latent factor of the students’ DC is composed by the six distinct DC factors.

Finally, to examine any significant differences among students across the DC components we conducted non-parametric statistical methods. We conducted a Mann–Whitney test to examine gender differences and Kruskal–Wallis tests to examine differences based on the students’ field of study and experience in computer use.

## Results

### Confirmatory factor analysis

The results of the PLS-SEM analysis suggest a good fit of the model on the values of NFI = 0.667 and Chi-Square = 843.442 according to the defined criteria of acceptance (Bryne, 2010; Hair et al., 2010; Kline, 2011). The value of Root Mean Square Error of Approximation (RMSEA = 0.088) indicated a score higher than 0.08 and less than 1.0 which is usually accepted as a good fit value, since a value of range between 0.05 and 1.00 are acceptable (Bandalos, 2018; Browne & Cudeck, 1992).

Also, the scores of the loading factors were highly valid (> 0.5) (Awang et al., 2010), and all the values of Cronbach alpha demonstrated internal consistency (Dijkstra & Henseler, 2015).

The bootstrapping results indicated that t (> 1.96) and p (< 0.01) values are all accepted and statistically significant. Composite reliability (CR) values indicate Internal consistency (Gefen et al., 2000) and average variance extracted (AVE) values indicate Convergent Reliability (Bagozzi & Yi, 1988; Chin, 2010; Fornell & Larcker, 1981), as depicted in Table 3.

**Table 3:** Reliability, validity, and internal consistency of the PLS-SEM measurement model

Components	Construct reliability (ρ_c_)			Average variance extracted (ρ_ν_)		
	Criteria^a^	Measurement	Interpretation	Criteria^a^	Measurement	Interpretation
SFA: Search, Find, Access	> 0.60	0.852	Highly reliable	> 0.5	0.537	Highly valid
DAM: Develop, Apply, Modify	> 0.60	0.867	Highly reliable	> 0.5	0.546	Highly valid
CCS: Collaborate, Communicate, Share	> 0.60	0.862	Highly reliable	> 0.5	0.677	Highly valid
SMD: Store, Manage, Delete	> 0.60	0.857	Highly reliable	> 0.5	40.527	Highly valid
EV: Evaluate	> 0.60	0.898	Highly reliable	> 0.5	0.594	Highly valid
PR: Protect	> 0.60	0.835	Highly reliable	> 0.5	0.628	Highly valid

^a^Muthén and Muthén (2012), Bandalos (2018)

As depicted in Table 4, the suggested students’ DC measurement model supports the discriminant validity between the constructs (Fornell & Larcker, 1981).

**Table 4:** Discriminant validity

	CCS	DAM	EV	PR	SFA	SMD
CCS	0.823					
DAM	0.608	0.724				
EV	0.652	0.721	0.771			
PR	0.601	0.605	0.689	0.792		
SFA	0.693	0.641	0.665	0.511	0.733	
SMD	0.727	0.639	0.686	0.608	0.703	0.739

A CB-SEM approach was also applied using the AMOS software and the maximum likelihood estimation to reinforce or compare the findings. The CB-SEM analysis validated the factor loadings of all items although indicating lower values. The approach revealed good results in terms of the fitness of the model: χ^2^/df = 2.02, Probability level = 0.000, RMSEA = 0.080. However, the comparative fit index (CFI) = 0.84, the Tucker–Lewis fit index (TLI) = 0.80 and revealed values slightly lower that the suggested thresholds or marginally accepted (Bandalos, 2018; Browne & Cudeck, 1992; Carmines & McIver, 1981; Hoyle, 1995; Muthén & Muthén, 2012).

Table 5 illustrates the unstandardized and standardized parameter estimates; as depicted, the critical ratio (C.R.) of constructs is more than 1.96 and all estimates are all statistically significant at the alpha level of 0.000 (Hair et al., 2010).

**Table 5:** Results of CB-SEM CFA of the 28-items SDiCoS students’ digital competence scale

		Unstandardized estimate	Standardized estimate	Standard error	C.R.^a^	p-value	Label
SFA1		4.538	0.578	0.054	83.891	***	Accepted
SFA2		4.135	0.605	0.069	60.17	***	Accepted
SFA3		4.263	0.642	0.064	66.883	***	Accepted
SFA4		4.295	0.752	0.078	54.816	***	Accepted
SFA5		4.244	0.78	0.066	64.476	***	Accepted
DAM1		3.628	0.637	0.096	37.828	***	Accepted
DAM2		2.231	0.587	0.102	21.865	***	Accepted
DAM3		3.686	0.805	0.089	41.192	***	Accepted
DAM4		1.897	0.582	0.091	20.741	***	Accepted
DAM5		2.558	0.639	0.097	26.312	***	Accepted
DAM6		4.122	0.670	0.88	46.909	***	Accepted
CCS1		4.699	0.787	0.049	95.252	***	Accepted
CCS2		3.654	0.630	0.087	41.965	***	Accepted
CCS3		4.687	0.764	0.052	90.997	***	Accepted
SMD1		4.096	0.670	0.081	50.744	***	Accepted
SMD2		4.596	0.526	0.057	80.128	***	Accepted
SMD3		4.795	0.586	0.039	121.860	***	Accepted
SMD4		4.737	0.679	0.041	116.146	***	Accepted
SMD5		4.321	0.725	0.071	60.832	***	Accepted
EV1		4.000	0.711	0.073	54.977	***	Accepted
EV2		4.199	0.728	0.069	60.925	***	Accepted
EV3		4.006	0.678	0.069	58.096	***	Accepted
EV4		3.833	0.773	0.080	48.086	***	Accepted
EV5		3.865	0.703	0.091	42.273	***	Accepted
EV6		3.846	0.698	0.087	44.054	***	Accepted
PR1		4.122	0.725	0.088	46.909	***	Accepted
PR3		4.115	0.578	0.083	49.680	***	Accepted
PR3		3.462	0.713	0.092	37.433	***	Accepted

*** Means p-value is significant in AMOS output

^a^Critical ratio (C.R.) of constructs is more than 1.96 and standardized estimates are significant (Hair et al., 2010)

A second order CFA analysis was finally conducted via the AMOS software. The results indicated a good fit of the SDiCoS model (Bandalos, 2018; Muthén & Muthén, 2012). RMSEA = 0.80, χ^2^/df = 2.04 and the p value is significant (p-value = 0.00). However, the increment fit indices (TFI = 0.84) show values below 0.9 and Hoelter values are below 200 indicating unsuitability of the sample size, mainly for a CB-based approach. Although CFI is not below 0.8 (= 0.83), it is accepted since “a value less than 0.10 or of 0.08 (in a more conservative version)” is a good fit of the model (Hu & Bentler, 1999). Overall, we can conclude that both the first and the second-order CBA models are generally considered as much valid as the PLS-SEM model that appears to indicate strong validity and reliability scores.

### Student differences across the SDiCoS components

This study also examined the potential differences in students’ groups according to (i) gender, (ii) age, (iii) field of study (Programme) and (iv) experience of computer use, across mean scores across the six DC constructs, as defined in RQ2.

Interestingly, gender showed no significant differences. This finding agrees with recent reports regarding the digital skills of young adult females and males across Europe (European Commission, 2019b) although there is contradictory evidence as well (e.g., in He & Zhu, 2017). Moreover, since previous studies (e.g., Burnett et al., 2010; Terzis & Economides, 2012; Tzafilkou et al., 2016) revealed significant gender differences in perception and acceptance towards computer-related tasks, this study results are encouraging to the future of the worldwide endeavor to eliminate the permanently existing gender gap in computing (European Commission, 2018b). However, similar studies in secondary education students (Hinostroza et al., 2015) reveled no gender differences in computer related learning skills.

As presented in Table 6, age revealed one significant correlation (p < 0.05) with the factors of SMD and PR. Students between 25 and 35 revealed the highest levels in both constructs, while the youngest team of 18–24 expressed the lowest scores. This result implies that undergraduate students meet difficulties, or they lack the skills in protection and file management tasks and renders serious consideration since according to Eurostat (2020) younger Europeans (20–24) tend use Internet, text, and multimedia much more frequently than older groups (25–64), however they might lack some essential ‘out-of-Internet’ or ‘out-of-social-media’ skills like file management and file/data protection.

**Table 6:** Kruskal Wallis tests on student groups across the six components of SDiCoS students’ digital competence scale

	SFA	DAM	CCS	SMD	EV	PR
Grouping variable: Age						
Chi-square	5.767	7.357	5.074	7.846	7.713	10.26
df	3	3	3	3	3	3
Asymp. sig	0.124	0.061	0.166	0.049*	0.052	0.016*
Grouping variable: Field of study						
Chi-square	9.378	11.371	10.275	11.768	9.391	12.861
df	4	4	4	4	4	4
Asymp. sig	0.052	0.023*	0.036*	0.019*	0.052	0.012*
Grouping variable: Computer experience						
Chi-square	2.573	4.991	7.543	16.59	4.949	5.447
df	3	3	3	3	3	3
Asymp. sig	0.462	0.172	0.056	0.001*	0.176	0.142

*Correlation is significant at the 0.05 level (2-tailed)

Although computer experience was significantly correlated to only component (SMD), the field of study showed several significant correlations in the components of DAM, CCS, SMD and PR. The post-graduate students in Digital Marketing expressed the highest scores across all the DC components, while the undergraduate programme of e-Commerce and e-Business showed the lowest values. However, most of the postgraduate students participated in the survey during the COVID-19 crisis, hence future research should examine whether this situation affected their responses and caused the difference in the groups-compared results.

Overall, the comparative results of this study can be generalized since the participants reflect a representative sample of higher education students in Greece, in terms of gender and age. Moreover, portions of different programmes (undergraduate and postgraduate) are considered in the study. However, different programmes (e.g., in different fields) or different regions might encounter significant differences in the students’ characteristics. Hence more research should be conducted on different student population to reinforce and validate the findings.

## Discussion, implications, and limitations

The main objective of this study (RQ1) was to measure and validate SDiCoS, a new students’ digital competence scale encompassing several digital skills essential to the pre, during and post-pandemic context of ERE. The suggested model was based on a comprehensive instrument and framework designed by Perifanou and Economides (2019a, 2019b) which was informed by and extended previous DC frameworks (DIGCOMP, UNESCO, ESDF, ESCO, ICILS, etc.). The resulting six-factor and 28 items scale has been validated using a hybrid CFA approach combining SEM-PLS with CB-SEM CFA approaches and using SmartPLS and AMOS software. Results indicate that the PSL-SEM CFA produced valid values of construct validity and reliability and accepted model fit criteria, while the CB-SEM approach revealed a similar fit to the model (RMSEA = 0.08), but it scored lower factor loadings. These findings are in accordance with Asyraf and Afthanorhan (2013) who explained this issue via augmenting that PLS-SEM is more appropriate for CFA for not normally distributed data.

Compared to previous quantitative studies (e.g., Alarcón et al., 2020; Kong et al., 2019; Peart et al., 2020; Suwanroj et al., 2019; Touron et al., 2018; etc.) the present study is the only one presenting a hybrid approach where both PLS-SEM and CB-SEM approaches are implemented for CFA of scale validation.

Furthermore, contrary to previous studies that suggested too short (e.g., Lee et al., 2015) or quite long (e.g., Peart et al., 2020; Touron et al., 2018) instruments, the SDiCoS proposes a comprehensive model of six components and 28 items, providing a practical and easy to use instrument for future research on students’ DC. SDiCoS includes all the essential components as derived from previous popular frameworks, being adjusted to the present technological trends. SDiCoS is a validated scale of students’ digital competence that considers all six important skills components: (1) Search, Find, Access (SFA); (2) Develop, Apply, Modify (DAM); (3) Communicate, Collaborate, Share (CCS); (4) Store, Manage, Delete (SMD); (5) Evaluate (EV); and (6) Protect (PR).

Previous scales on students’ digital competence either ignore important components such as ‘Protect’ (e.g., Elstad & Christophersen, 2017; Koc & Barut, 2016; Lau & Yuen, 2014; Lee et al., 2015; Siddiq et al., 2016; Suwanroj et al., 2019), or take a completely different approach by considering components such as “parental ICT attitude” (Aesaert et al., 2015), “language integration” (Hatlevik et al., 2015), “Internet political activism” (Choi et al., 2017).

Furthermore, only few scales have been validated for undergraduate university students (Elstad & Christophersen, 2017; Koc & Barut, 2016; Kuzminska et al., 2018; Lee et al., 2015; Suwanroj et al., 2019), and postgraduate university students (Choi et al., 2017).

Finally, the current quantitative study is the only one (among the scale development and validation studies) carried out in a South European country (Greece) focusing on higher education students’ digital skills. Thus, the current study seeks to contribute to the students’ DC awareness across different regions and towards the design of homogenous students’ DC scales worldwide.

The SDiCoS scale is useful to reveal skill polarities and gaps in the students DC among the examined components. For example, as described in the results, younger students expressed lower perceived skills in protection and file management tasks, although they are more actively engaged in Internet and social media activities, compared to older age-groups of students.

SDiCoS would be useful to the following stakeholders:

- Policymakers and decision-makers at national, and international levels who are responsible for taking strategic decisions for education, digital technologies, employment, economy, etc.;
- Directors of formal and continuing education institutes who work on setting goals, measuring, providing training and certification regarding their students’ digital competence;
- Educators at educational institutes who design curriculum and syllabus for formal and informal training;
- Teachers, in service and in training, who would improve their digital competence and integrate digital technologies in their teaching practice;
- Teachers who would become aware of their students’ digital competence needs, and take appropriate actions;
- Researchers on the use of digital technologies, on individuals’ digital competence and digital skills.
- Instructional designers and educational institutions that plan to trace their teaching and learning strategies in the context of blended and online learning.

For example, SDiCoS could help policymakers who aim to identify students’ digital competence level to:

- Design and organize educational adjustments and reforms, such as the emergent shift to remote education during the COVID-19 times or adjustments needed to the soft transition and/or maintenance to blended and online learning. In order to successfully design this shift, policymakers should know the level of students' (and teachers) digital competence (among other issues);
- Design and financially support massive and specialized training on digital technologies to fight discrimination, digital divide, and non-inclusion of citizens with low digital competence, and boost innovation, employability, participation in the digital market, and digital society (e.g., e-commerce, e-banking, e-government). Although 82% of European individuals 16 to 24 years old have basic or above basic overall digital skills, only 60% of European individuals 25 to 64 years old have such skills (Eurostat, 2020). The suggested SDiCoS can be used to design short-term sessions or extra ICT training when needed, to assist young and older students in acquiring all the basic digital skills that they potentially lack.

Furthermore, the validated SDiCoS can serve internally, as a practical and useful tool to evaluate the students’ perceived digital competence in higher and continuing education institutions, including their knowledge and skills on recent technological trends like social media and mobile use.

One main limitation of this study is the small sample size for the CFA. Although, the sample size is efficient for the PLS-SEM approach, further research in encouraged on larger populations in the future. Also, the COVID-19 crisis emerged during the collection of the response. This situation has might affect the responses of the students that responded remotely duo to the school closure and further research should be conducted to explore the role of COVID-19 on the students’ DC perceived items. Furthermore, this study examined any DC’s differences with respect to gender, age, field of study and computer experience. Future researcher should investigate other factors that may affect DC or examine ERE specific components like skills in remote synchronous collaboration, and text-based online learning. Finally, it would be interesting to conduct future research at a later stage of the pandemic, to examine how and whether the students’ DCs have been improved.

## Conclusions

This study develops and validates the SDiCoS scale to measure students’ digital competence. The proposed scale takes into consideration recent technological trends and previous studies on DC frameworks and provides the conceptual basis for understanding the main DC components in the context of remote education. The generated six-factor scale is composed of the following DC components: (1) Search, Find, Access; (2) Develop, Apply, Modify; (3) Communicate, Collaborate, Share; (4) Store, Manage, Delete; (5) Evaluate; and (6) Protect.

Regarding RQ1, the validity of SDiCoS was tested through both PLS-SEM and CB-SEM methods. The PLS-SEM based CFA approach demonstrated the SDiCoS validity, resulting in highly valid consistency and reliability, and accepted model fit criteria. A CB-SEM replication of the CFA and a second-order CFA was also conducted to complement, compare, and reinforce the findings.

Regarding RQ2, the statistical analysis indicated significant differences across the SDiCoS constructs between different groups of students, including their age, field of study and computer experience.

The SDiCoS model is usable for both undergraduate and post-graduate students in higher education and can be used to measure the students’ digital competence across the main DC components, concerning the recently emerged technological trends like remote/online education, social media, smart devices, mobile and safety skills.

## Data Availability

The datasets used and/or analysed during the current study are available from the corresponding author on reasonable request.

## Appendix

See Table 7.

**Table 7:** SDiCoS instrument: components of the students’ digital competence scale (SDiCoS) and measured items

Component	Acronym	Items	Acronym
1. Search, Find, Access	SFA	I can search and find a specific object or similar objects using various search engines (e.g., Google, Yahoo, Bing) and databases, using appropriate keywords and advanced criteria and filters	SFA1
		I can search and find a specific person on various social networks using various techniques and filters (e.g., various formats of name, photo, email address, school, company, etc.)	SFA2
		I can search and find groups on a specific topic (e.g., hobby, profession, artist, science, historical event, travel destination) on various social media	SFA3
		I can navigate in the real-world using the advanced features of a navigator	SFA4
		I can watch (read, listen, view) content in various formats on various smart devices	SFA5
2. Develop, Apply, Modify	DAM	I can create an event and set notifications using a digital calendar (e.g., Google Calendar, Apple Calendar, Microsoft Outlook Calendar)	DAM1
		I can creatively design and/or develop a website using various digital tools (e.g., Wix, WordPress)	DAM2
		I can create a document with text, diagrams, tables, reports, and advanced formatting	DAM3
		I can apply Creative Commons licenses to content or software that I have created	DAM4
		I can apply statistical techniques using appropriate software (e.g., SPSS, R, MS Excel, Google Sheets) to make forecasting or predictions	DAM5
		I can convert content from one format to another format	DAM6
3. Communicate, Collaborate, Share	CCS	Ι can collaborate with people using various smart devices, platforms, and digital tools	CCS1
		Ι can teach an e-course or an e-seminar, give a lecture or make a presentation using various digital tools	CCS2
		I can upload and share software or app that I have developed on various social media	CCS3
4. Store, Manage, Delete	SMD	I can take a photo or a video and save it in various formats (mp4, wmv, avi, qt, gif, jpg, etc.) using various smart devices and digital recording tools	SMD1
		I can download content and save it directly to the relevant folder	SMD2
		I can copy and save the screenshot from various smart devices	SMD3
		I can delete some of my connections/friends in various social networks	SMD4
		I can organize the files on my computer into a hierarchical folder structure	SMD5
5. Evaluate	EV	I can evaluate an object and/or a smart device using appropriate quality criteria (e.g., authenticity, utility, easy to use, appearance, functionality, enjoyment)	EV1
		I can critique an object and/or a smart device on relevant social media (e.g., TripAdvisor, YouTube, Amazon)	EV2
		I can evaluate whether some information is hoax, fake, scam, or fraud	EV3
		I can evaluate whether a website is secure and trusted	EV4
		I can identify the intellectual property rights (IPRs) of content that I have found on Internet	EV5
		I can evaluate whether an email is spam, adware, phishing, or fraud	EV6
6. Protect	PR	I can regularly change my passwords and settings of my smart devices and Internet accounts	PR1
		I can protect various smart devices and e-accounts using different passwords and frequently changing them	PR2
		I can protect myself and others against identity theft, harassment, bulling, or slander	PR3

All the items are measured on a 5-point Likert scale (1: strongly disagree to 5: strongly agree). Used terms explained to participants: Smart device = smartphone, tablet, laptop, pc, camera, navigator, game console, smart TV, etc.; Object = document, picture, movie, software, app, etc.
